# Cross species review of the physiological role of d-serine in translationally relevant behaviors

**DOI:** 10.1007/s00726-023-03338-6

**Published:** 2023-10-13

**Authors:** Dena Arizanovska, Jada A. Emodogo, Anna P. Lally, Caroline B. Palavicino-Maggio, Daniel J. Liebl, Oluwarotimi O. Folorunso

**Affiliations:** 1 The Miami Project to Cure Paralysis, Department of Neurosurgery, University of Miami Miller School of Medicine, Miami, FL USA; 2 Department of Psychiatry, Harvard Medical School, Boston, MA USA; 3 Translational Psychiatry Laboratory, McLean Hospital, Belmont, MA USA; 4 Neurobiological Mechanisms of Aggression Laboratory, McLean Hospital, Belmont, MA USA; 5 Translational Neuroscience Laboratory, McLean Hospital, Belmont, MA USA

**Keywords:** d-Serine, Serine racemase, Sleep, Cognition, Sociability

## Abstract

Bridging the gap between preclinical models of neurological and psychiatric disorders with their human manifestations is necessary to understand their underlying mechanisms, identify biomarkers, and develop novel therapeutics. Cognitive and social impairments underlie multiple neuropsychiatric and neurological disorders and are often comorbid with sleep disturbances, which can exacerbate poor outcomes. Importantly, many symptoms are conserved between vertebrates and invertebrates, although they may have subtle differences. Therefore, it is essential to determine the molecular mechanisms underlying these behaviors across different species and their translatability to humans. Genome-wide association studies have indicated an association between glutamatergic gene variants and both the risk and frequency of psychiatric disorders such as schizophrenia, bipolar disorder, and autism spectrum disorder. For example, changes in glutamatergic neurotransmission, such as glutamate receptor subtype *N*-methyl-d-aspartate receptor (NMDAR) hypofunction, have been shown to contribute to the pathophysiology of schizophrenia. Furthermore, in neurological disorders, such as traumatic brain injury and Alzheimer’s disease, hyperactivation of NMDARs leads to synaptic damage. In addition to glutamate binding, NMDARs require the binding of a co-agonist d-serine or glycine to the GluN1 subunit to open. d-serine, which is racemized from l-serine by the neuronal enzyme serine racemase (SRR), and both SRR and d-serine are enriched in cortico-limbic brain regions. d-serine is critical for complex behaviors, such as cognition and social behavior, where dysregulation of its synthesis and release has been implicated in many pathological conditions. In this review, we explore the role of d-serine in behaviors that are translationally relevant to multiple psychiatric and neurological disorders in different models across species.

## Introduction

Finding effective treatments for psychiatric and neurological disorders depends partly on developing animal models with validated mechanisms that are translatable across species. Cognitive and social deficits are core components of multiple neuropsychiatric and neurodegenerative conditions (Stuchlik & Sumiyoshi 2014), while sleep disruption is commonly observed in many disorders, including anxiety, depression, and schizophrenia. Furthermore, sleep is involved in many processes such as memory consolidation, emotional stability, and the maintenance of brain homeostasis, where its dysregulation could have a bidirectional effect on symptom progression and outcomes (Sun et al. 2022). These behaviors are conserved among vertebrates and invertebrates (such as rodents and fruit flies, respectively), making them valuable models for studying human pathophysiology. Therefore, it is important to identify common factors underlying the molecular mechanisms of these behaviors.

Genome-wide association studies have implicated glutatamertegic gene variants in both the risk and frequency of psychiatric disorders like schizophrenia, biopolar disorder, and autism spectrum disorder (ASD) (Singh et al. 2022; Trubetskoy et al. 2022). Furthermore, preclinical and clinical studies have shown that receptors important for glutamatergic neurotransmission, such as the glutamate receptor subtype *N*-methyl-d-aspartate receptors (NMDARs), contribute to the pathophysiology of neurodegenerative and neuropsychiatric disorders (Singh et al. 2022; Wang & Reddy 2017). NMDARs are unique ligand-gated ion channels that require the binding of glutamate at the GluN2 subunit as well as a co-agonist, glycine or d-serine, at the GluN1 glycine modulatory site (Wolosker et al. 1999a, b). In corticolimbic areas that are critical for cognitive and social behaviors, such as the prefrontal cortex, nucleus accumbens, hippocampus, and amygdala, d-serine parallels the expression pattern of and is the preferred co-agonist of NMDARs (Dong et al. 2018; Hashimoto et al. 1993; Matsui et al. 1995; Mothet et al. 2000; Schell et al. 1997, 1995; Shleper et al. 2005). d-serine is also expressed in the hypothalamus (Chieffi Baccari et al. 2020), where the role of NMDARs, in addition to traditional monoaminergic pathways, has been increasingly appreciated in the regulation of sleep and wakefulness (Saper & Fuller 2017). It is important to mention that glycine could also play a complementary role that should be further investigated (Meunier et al. 2016; Papouin et al. 2012; Stroebel et al. 2021).

d-Serine is converted from l-serine by the neuronal enzyme serine racemase (SRR) (Wolosker et al. 1999a, b). During development, there is a spatiotemporal increase in SRR expression in corticolimbic regions, suggesting a role for D-serine in proper circuit formation (Folorunso et al. 2021). Furthermore, changes in synaptic plasticity, such as NMDAR-dependent long-term potentiation (LTP), are critical for memory formation, particularly in the adult hippocampus and amygdala of mice (Balu et al. 2013; Basu et al. 2009; Le Bail et al. 2015; Li et al. 2013; Papouin et al. 2012). We have shown that D-serine is postsynaptically released to regulate synaptic NMDAR function at Schaffer collateral CA3-CA1 synapses (Wong et al. 2020), where genetic ablation of neuronal SRR leads to deficits in hippocampal LTP (Perez et al. 2017). Recent large-scale genome-wide association studies have identified a functional single nucleotide polymorphism in the *SRR* gene associated with schizophrenia (Pardinas et al. 2018; Schizophrenia Working Group of the Psychiatric Genomics 2014), highlighting its relevance to psychiatric disorders. Additionally, lower levels of d-serine and SRR have been reported in the brains and serum of people with schizophrenia (Hashimoto et al. 2003). Under pathological conditions, such as Alzheimer’s disease (AD) and traumatic brain injury (TBI), we show that d-serine production is upregulated by reactive glial cells and may contribute to excitotoxicity and neurodegeneration (Balu et al. 2019; Folorunso et al. 2023; Perez et al. 2017; Tapanes et al. 2022). Together, these studies highlight a cell-specific role for d-serine in learning and memory, and demonstrate that dysregulation of d-serine can lead to alterations in behavior.

Rodent models and invertebrate organisms are valuable for understanding how genes and neural circuits control behavior due to the large number of tools available for genetic manipulation. *Drosophila melanogaster* (fruit flies), are particularly important for studying the neurological and genetic underpinnings of sleep, wakefulness, and cognitive tasks (Grover et al. 2022; Koh et al. 2008; Liu et al. 2008). Using olfactory and visual cues, fruit flies have been used to study associative learning through both delay and trace conditioning paradigms (Grover et al. 2022), as well as decision-making via feeding assays (Yu et al. 2021). Similar to humans and rodents, fruit flies follow a circadian rhythm where they sleep on a 12 h light/dark cycle that is influenced by variables including feeding, stress, and social environments(Beckwith & French 2019; Hendricks et al. 2000; Nakagawa et al. 2022). Moreover, an age-related decline in associative memory has been observed in fruit flies, mirroring that observed in rodents and humans (Konig & Gerber 2022). Further similarities across organisms exist, as it has also been shown that flies exhibit social behaviors such as aggression and courtship (Bentzur et al. 2021; Soto-Yeber et al. 2018), as well as changes in motivation, experience, activity level, and sensory sensitivity after social isolation (Soto-Yeber et al. 2018).

Rodents are commonly used to study social behavior, where intricate circuitry between the prefrontal cortex and subcortical areas, including the amygdala, nucleus accumbens, ventral tegmental area, hypothalamus, and hippocampus, is involved (Bicks et al. 2015). While rodents possess a less developed prefrontal cortex and a greater proportion of olfactory input (Wei et al. 2021), homology between the circuitry underlying human and rodent social behavior has been observed. For example, connections between the medial prefrontal cortex and amygdala are altered during social interactions and are significantly different in models of autism spectrum disorder (Kuga et al. 2022). Furthermore, while rodent models cannot recapitulate the entirety of human social behavior, some aspects, such as social interaction and withdrawal, have been well-characterized and widely validated for the study of neuropsychiatric disorders. For instance, asociality is a prominent negative symptom of schizophrenia that is commonly studied in mice using the three-chamber interaction test (Ellenbroek & Cools 2000; O'Tuathaigh et al. 2014, 2009). Moreover, behaviors during free dyadic social interactions (Kraeuter et al. 2019), as well as ultrasonic vocalizations (USVs), can be used to measure communicative behavior in various social contexts and neurodevelopmental disorders (Lefebvre et al. 2020; Premoli et al. 2021; Sangiamo et al. 2020; Scattoni et al. 2009; Seffer et al. 2014). In addition, rodents are widely used to study cognition, where spatial memory assays and passive avoidance testing are well-validated measures of learning and memory. Moreover, the use of rodents in contextual fear learning assays allows an in-depth understanding of various factors (genetics, stress, drug effects, etc.) that contribute to cognitive and behavioral responses to stress and threat. In addition, their small size, ease of handling, and well-characterized sleep patterns enable the study of mechanisms underlying sleep and the effects of sleep deprivation. Like humans, rodents experience non-rapid eye movement (NREM) and rapid eye movement (REM) sleep, and a sleep–wake cycle controlled by the interaction of several brain regions and neurotransmitter systems (Aguilar et al. 2021). While rodents have shorter sleep periods than humans and spend different proportions of time in NREM versus REM sleep (Kawai et al. 2015), studies have shown that sleep deprivation impairs learning and memory and leads to changes in brain structure and function in rodents, which is similar to what is observed in humans (Colavito et al. 2013).

This review focuses on the role of d-serine in physiologically relevant behaviors across different species, including fruit flies, rodents, and humans, and highlights the importance of d-serine in sleep, cognition, and social interactions. However, further cross-species studies in various preclinical models and clinical settings are necessary to validate the role of d-serine in these behaviors.

### d-Serine and cognitive tasks

d-Serine has been shown to be important for attention, motivation, learning, and memory behaviors across invertebrates to humans. Genetic mutations, d-serine administration, NMDAR antagonists, and stress models have been used to determine how d-serine alters cognitive performance.

#### Fruit flies

In genetically modified flies, olfactory-based behavioral assays, such as the shock-paired odor conditioning test, were used to determine the effect of d-serine on learning and memory. In this assay, adult flies were initially exposed to an odor paired with an electric shock (conditioned stimulus, CS) and to a second odor without an accompanying shock. After this initial training, wildtype (WT) flies learned to avoid the CS when given a choice in subsequent behavioral assays. However, aged WT flies or young flies overexpressing drosophila pyruvate carboxylase (dPC), an enzyme that normally increases with age, display several types of memory impairments in the shock-paired odor conditioning test (Yamazaki et al. 2014) (Table 1). Both aging and dPC overexpression led to a decrease in the ratio of d- to l-serine, where the administration of d-serine through feeding reversed learning and memory deficits.

**Table 1:** Effect of d-serine on cognition

Model	Organism	d-serine levels	Cognition/behavioral outcome
WT (aged) (Yamazaki et al. 2014)	Drosophila	↓	Shock-paired odor memory task (SOMT); decreased memory retention of negative stimulus in aged WT flies given T maze odor discrimination task, + D-serine (1 mM) rescued behavior
Overexpressing pyruvate carboxylase (dPC +) (Yamazaki et al. 2014)	Drosophila	↓	SOMT; decreased memory retention of negative stimulus in dPC + flies given T maze odor discrimination task, + D-serine (1 mM) rescued behavior
WT (Balu et al. 2018)	Mice	↑ amygdala	D-serine is increased after fear conditioning, D-serine (300 mg/kg) administration facilitated acquisition and retention of exticition
Acute restraint stress (Guercio et al. 2014)	Mice	↓ Prefrontal cortex	Object recognition task; novel object exploration decreased in stressed animals due to effect of acute stress on memory consolidation, + D-serine (1 g/kg, i.p.) rescued behavior
SRR ENU mutagenesis D-serine (SrrY^269*^) (Labrie, 2009)	Mice	↓	Spatial change session of the object discrimination task; increased time spent with displaced objects in WT mice, no difference in exploratory behavior of displaced objects versus stationary objects in mutant mice, + D-serine (600 mg/kg) rescued behavior
SRR ENU mutagenesis D-serine (Labrie et al. 2009a, b)	Mice	↓	Morris Water Maze (MWM); decreased time spent in target area during swim test + D-serine (600 mg/kg) rescued behavior Novel object recognition test no significant difference in object exploration time
SRR KO (DeVito et al. 2011)	Mice	↓	Order task: exhibited an opposite pattern of preference for the order of events in distinct experiences in object or odor tests
SRR KO (DeVito et al. 2011)	Mice	↓	Object displacement task; spend more time exploring a recently experienced object than a previous object which was opposite to WT
SRR KO (DeVito et al. 2011; Matveeva et al. 2019)	Mice	↓	Novel object recognition test no significant difference in object exploration time
SRR KO (Basu et al. 2016)	Mice	Not shown	Decreased freezing associated with impaired contextual fear memory
SRR KO (Inoue et al. 2018)	Mice	Not shown	No change in freezing showing impairment in fear extinction; Reduction in freezing showing decreased fear memory retrieval
CX_3_CR_1_^creErt2^:SRR^fl/fl^ GFAP^creErt2^:SRR ^fl/fl^ TMEM119:SRR ^fl/fl^ CX_3_CR_1_^creErt2^:Slc1a4^fl/fl^ GFAP^creErt2^:Slc1a4 ^fl/fl^ (Perez et al. 2017; Tapanes et al. 2022)	Mice	↓ (in glia)	Prevents impairments in contextual fear conditioning after CCI
WT (Aged; 18–20, 22–24 mo.) (Nava-Gomez et al. 2022b)	Rat	Not shown	Reversal learning task; decreased cognitive flexibility + D-serine (300 mg/kg) rescued behavior
ICV injection of Aβ (Nikseresht et al. 2021)	Rat	Not shown	Passive avoidance task; decreased avoidance post-conditioned foot shock-paired stimulus + (2/5 μmol /4 μl D-cycloserine) rescued behavior
NMDAR antagonist: MK- 801 (Nagy et al. 2021)	Rat	Not shown	Passive Avoidance task; decreased avoidance post-conditioned foot shock-paired stimulus + D-serine (640 mg/kg) or DAAO inhibitor (CPD30; 0.1 mg/kg, prevents D-serine degradation) rescued behavior
WT (adult) (Bai et al. 2014)	Rat	Not shown	Inhibitory Avoidance Task; latency to enter dark/shock-paired compartment + D-serine (800 mg/kg) enhanced extinction learning
Humans with Schizophrenia (Hons et al. 2021)	Humans	↓ Serum	Participants scoring lower on Rey–Osterrieth Complex Figure (learning and memory), Trail Making (attention, processing speed, visual-motor coordination), and Wisconsin Card Sorting (problem solving and reasoning) tests showed a lower average serum level of D-serine and D-serine/total serum ratio
Humans with Post traumatic stress disorder (PTSD) (Inslicht et al. 2022)	Humans	Not shown	+ D-cycloserine (50 mg) group demonstrated decreased Skin Conductance Response (Extinction learning) and a trend towards decreased Skin Conductance Response extinction retention

*WT* wild-type, *SRR KO* serine racemase knockout, *RLT* reversal learning task, *PAT* passive avoidance task, *ORT* object recognition task, –*OMT* shock-paired odor memory task, *DAAO* d-Amino Acid Oxidase

#### Rodents

Many studies have employed genetically modified SRR mice to assess the role of d-serine levels in cognitive function. To assess episodic memory, WT and germline SRR knockout mice were evaluated in two sequence memory tasks for their ability to remember the order in which specific objects or odors were presented (DeVito et al. 2011). On both assays, SRR knockout mice expressed opposite order preference behavior from WT mice. This indicates that mice lacking d-serine are able to discriminate and remember the temporal order of events, but their memory expression is impaired. Interestingly, SRR knockout mice had reduced branching, length, and spine density in apical dendrites of the medial prefrontal cortex, suggesting a possible disruption in the hippocampal-medial prefrontal cortex circuitry that could bias mice lacking d-serine to more recent memory events. Conversely, no differences between SRR knockout and WT mice were found on object recognition and displacement tasks, which assess preference for a novel versus familiar object and relocated versus stationary object, respectively (DeVito et al. 2011). This finding implies that d-serine is involved in specific aspects of cognition, such as the representation of event order, but not all aspects of learning and memory (DeVito et al. 2011). This finding was replicated in an object recognition test in another study using germline SRR knockout mice; however, they showed that mutant mice spent more time investigating the two identical objects during the acquisition phase of the test (Matveeva et al. 2019). This suggests that despite having intact long-term object recognition memory, d-serine may affect the learning process, as SRR knockout mice may require more time to establish a stable memory of these objects. In another model employing *N*-nitroso-*N*-ethyl urea (ENU) mutagenesis, in which point mutations were introduced into the genome, resulting SRR^Y269*^ mutant mice that lack SRR activity also showed no deficit in object recognition (Labrie et al. 2009a, b). Similar to SRR knockout mice, SRR^Y269*^ mutants did not show impaired performance in the novel object test (Labrie et al. 2009a, b). However, SRR^Y269*^ mice had a significant deficit in the object displacement task that could be rescued by subcutaneous d-serine administration (600 mg/kg) (Labrie et al. 2009a, b). It is important to note that it is difficult to determine whether this mutagenic approach is specific to SRR, as other genes are likely to be affected. Furthermore, the SRR^Y269*^ mutant study used male and female data compared to SRR knockout studies that used only males, which may contribute to some of the differences observed between groups in the object displacement test. An impairment was also observed in the Morris water maze, a spatial learning task where the latency to locate a hidden underwater platform was measured; this deficit was similarly rescued via exogenous d-serine administration (Labrie et al. 2009a, b). Unlike object recognition, the object displacement task evaluates spatial memory (Denninger et al. 2018). This may explain why similar deficits were observed on this assay as the Morris water maze and implies that d-serine plays a critical role in spatial learning and memory, which relies predominantly on the hippocampus. In another study employing germline SRR knockout mice, a similar deficit was observed in males but not females on the Morris water maze (Basu et al. 2009). As female rodents have been shown to engage the striatum in spatial learning tasks (Yagi and Galea 2019), this further suggests a preferential role of d-serine in hippocampal-dependent learning and memory. Furthermore, d-serine has been implicated in other hippocampal-dependent learning and memory assays, as SRR knockout mice showed decreased contextual fear memory on a trace conditioning assay (Basu et al. 2016). Supporting these findings, we have shown that neuronal SRR is required for hippocampal long-term potentiation (LTP), a process that underlies learning and memory (Perez et al. 2017). However, in a controlled cortical impact (CCI) mouse model of TBI, astrocyte and microglia cells upregulate d-serine production and release, contributing to cognitive impairments after TBI. Blocking the synthesis of glial d-serine rescued hippocampal synapses, LTP, and contextual fear memory after CCI (Perez et al. 2017; Tapanes et al. 2022). Furthermore, pharmacological blockade or genetic ablation of glial d-serine transporters Slc1a4 and Slc7a10 similarly protected against CCI-induced learning and memory impairments, illustrating the importance of studying cell-specific effects of d-serine on cognition (Tapanes et al. 2022).

Administration of d-serine in models of aging, stress, and NMDAR antagonist treatment rescued memory impairments in various learning tasks, including the Morris water maze (Table 1). In a reversal learning attention test measuring cognitive flexibility performance, d-serine supplementation via drinking water (300 mg/kg/day) normalizes the decline in cognitive flexibility observed in middle-aged (18–20 months) and aged (22–24 months) rats (Nava-Gomez et al. 2022a). Furthermore, d-serine prevents acute stress-induced impairments in memory consolidation in adult mice in the object recognition test (Guercio et al. 2014). In an AD mouse model using Aβ1–42 injection, D-serine administration rescued impairments in spatial memory on the Morris water maze and improved associative memory deficits on the passive avoidance learning task, which measures the latency to enter a compartment that had previously been paired with a negative stimulus (i.e., foot-shock) (Nikseresht et al. 2021). This mechanism is likely through d-serine activity at NMDARs, as administration of the NMDAR antagonist MK-801 induced memory impairments in rats on the passive avoidance task that were reversed by d-serine (640 mg/kg) (Nagy et al. 2021).

In an inhibitory avoidance task, which uses a foot shock as a negative stimulus, intraperitoneal administration of d-serine (800 mg/kg) an hour prior to extinction training accelerated fear memory extinction (Bai et al. 2014), while d-serine (2.7 g/kg,) administered ten minutes prior to retrieval training rescued deficits in extinction recall memory in SRR knockout mice (Inoue et al. 2018) (Table 1). Extinction of contextual fear memory was also facilitated in mice harboring the hypofunctional Dao1(G181R) mutation, in which the activity of DAAO, the enzyme that degrades d-serine, was inhibited, leading to higher d-serine levels (Labrie et al. 2009a, b). Moreover, trace fear conditioning impairments in SRR knockout mice are restored by systemic treatment with d-serine (300 mg/kg), while SRR and d-serine are dynamically regulated by fear conditioning and extinction in the mouse amygdala (Balu et al. 2013, 2018; Heresco-Levy et al. 2009; Walker et al. 2002; Wolosker & Balu 2020) (Table 1). Together, these studies highlight a critical role for SRR and d-serine in various aspects of cognition, where decreased levels of d-serine lead to impairments in hippocampal-dependent learning and memory.

#### Humans

Studies have shown the benefits of d-serine administration on cognition in humans; however, these studies become more complicated as they are add-on treatments to antipsychotics. Lower serum levels of d-serine and d-serine/total serine ratio were correlated to poor performance on executive function tasks, such as the Rey–Osterrieth Complex Figure, Trail Making, and Wisconsin Card Sorting tests (Hons et al. 2021). Moreover, Hons and colleagues summarized studies that showed the addition of d-serine (30–120 mg/kg) to ongoing antipsychotic or cognitive retraining (CRT) treatment in people with schizophrenia improved cognitive functions on various tasks (i.e. Wisconsin Card Sort Test (WCST), Measurement and Treatment Research to Improve Cognition in Schizophrenia (MATRICS) domains, Hopkins Verbal Learning Test-Revised (HVLT-R), WAIS-III Logical Memory, Tower of London (TOL) executive functioning task, and continuous performance test) (Hons et al. 2021) (Table 1). Conversely, in a 16-week trial of d-serine (2 g/day) as an add-on treatment to antipsychotics, there was no significant improvement in the MATRICS cognitive score (Weiser et al. 2012). In controlled trials, systemic administration of d-cycloserine, a partial agonist of the NMDAR GMS, which can increase extracellular levels of brain d-serine, reduced symptoms of acrophobia (fear of heights) (Ressler et al. 2004) and d-serine treatment reduced symptoms in people with post-traumatic stress disorder (PTSD) (Heresco-Levy et al. 2009). Furthermore, individuals with PTSD showed significantly enhanced fear extinction after taking d-cycloserine, as measured by skin conductance response, with a trend towards increased extinction retention. (Inslicht et al. 2022) (Table 1). It is important to note, however, that many of these clinical studies had small sample sizes and included subjects primarily with schizophrenia, PTSD, or dementia; more large-scale studies that include other disorders with cognitive decline are needed to fully understand the role of d-serine in cognition.

## d-Serine and social behaviors

d-serine plays a crucial role in social functioning, where its dysregulation has been implicated in neuropsychiatric conditions such as depression and schizophrenia (reviewed in Cho et al. 2016; de Bartolomeis et al. 2022; MacKay et al. 2019; Pei et al. 2021).

### Rodents

Rodent models of depression and schizophrenia commonly rely on the three-chamber sociability test to assess motivation for social interactions (Nadler et al. 2004). In this paradigm, animals are allowed to freely explore an arena with three compartments. The first phase of the test measures sociability, in which one chamber contains a rodent and the other a novel object. Time spent in each chamber is recorded, where rodents with higher sociability will spend more time with the other animal than an object. In the second phase of the test, the object is replaced with a novel animal, and time spent in the chamber with the original (now familiar) versus novel animal is evaluated to assess preference for social novelty (Crawley 2004; Kaidanovich-Beilin et al. 2011; Nadler et al. 2004). Using this assay, Matveeva and colleagues found that while there were no differences in the sociability phase of the test*,* germline SRR knockout mice spent more time investigating the familiar versus novel mouse as opposed to WT mice, implicating reduced d-serine in impaired preference for social novelty (Matveeva et al. 2019). A recent study by Aguilar and colleagues found similar results, as both WT and SRR knockout mice preferred exploring a novel mouse versus a novel object (Aguilar et al. 2021). However, in the social novelty phase, WT mice spent a significantly greater proportion of time, as well as had more entries, into the chamber with the novel mouse than with the familiar mouse. Interestingly, EEG recordings revealed that SRR knockout mice had reduced low gamma power in the frontal cortex at the onset of investigating the novel mouse, as well as enhanced background gamma during the task. As people with schizophrenia have impaired frontal cortex gamma power, likely due to GABAergic interneuron dysfunction and cortical disinhibition, this study demonstrates a link between d-serine, social behavior, and schizophrenia-like phenotypes (Aguilar et al. 2021).

Rather than genetic knockout, Labrie and colleagues employed ENU mutagenesis to introduce spontaneous point mutations into the *Srr* genome (Labrie et al. 2009a, b) (Table 2). They observed an approximately 50% reduction of *Srr* mRNA, resulting in significant protein loss and no d-serine production within the whole brain. Contrary to SRR knockout mice, SRR^Y269*^ mice expressed deficits in the sociability but not social novelty phase of the three-chamber interaction test, as they spent more time exploring the object rather than the novel animal. This discrepancy could be due to the use of different models of SRR mutagenesis, where ENU mutagenesis has greater off-target effects than direct genetic knockout. Even so, these studies collectively highlight the important role of SRR and d-serine synthesis in social interactions. In further support of this, the administration of d-serine was sufficient to restore social behavior in SRR^Y269*^ mice (Labrie et al. 2009a, b) (Table 2). Only one study found no effect of SRR knockout on the three-chamber interaction test (DeVito et al. 2011); however, this may be due to the use of a littermate as the stimulus mouse, thus masking any potential deficits in sociability that would be present with a stranger mouse, as used in the other studies.

**Table 2:** Effect of d-serine on social behavior

Model: genetic/treatment	Organism	d-serine levels	Social behavior
SRR KO (DeVito et al. 2011)	Mice	↓	Three chamber sociability test; no changes in sociability or preference for social novelty
SRR KO (Matveeva et al. 2019)	Mice	↓	Three chamber sociability test; no difference in sociability deficit in social novelty preference
SRR KO (Aguilar et al. 2021)	Mice	↓	Three chamber sociability test; no difference in sociability deficit in social novelty preference EEG recordings; decreased low gamma power in frontal cortex during onset of social investigation
SRR ENU mutagenesis: SrrY^269*^ (Labrie et al. 2009a, b)	Mice	↓ In whole brain, hippocampus frontal cortex	Three chamber sociability test; deficit in sociability no change in preference for social novelty + D-serine rescued sociability
WT (control and CSDS), bilateral hippocampal D-serine or ASCT2 shRNA injection (Wang et al. 2017)	Mice	↓ HP	Social interaction test; decreased time spent in social interaction zone + D-serine or ASCT2 knockdown rescued behavior
*Grin1*^*D481N*^ mutant *Grin1*^*D481N*^ and *Daao*^*G181R*^ double mutant (Labrie et al. 2010)	Mice	N/A in *Grin1*^*D481N*^, ↑ double mutant WB	Three chamber sociability test; deficits in sociability and preference for social novelty Double mutation rescued behavior
Grin1 hypomorph, (A. N. Hanks et al. 2013a, b)	Mice	Not shown	Three chamber sociability test; deficit in sociability + D-serine had no effect on sociability
Balb/c, IP D-serine injection (Jacome et al. 2011)	Mice	Not shown	Three chamber sociability test; deficit in sociability + D-serine (560 m/kg IP) rescued sociability
PolyI:C injection, IP D-serine injection (Nagai et al. 2012)	Mice	Not shown	Social interaction with intruder decreased + D-serine (1.0 g/kg IP) rescued behavior
NLG3 knockin (Cao et al. 2022)	Mice	Not shown	Three chamber sociability test; deficit in social novelty preference + D-cycloserine (IP or prefrontal cortex infusion) rescued behavior
Valproic acid-exposure (Wu et al. 2018)	Rats	Not shown	Three chamber sociability test; deficit in + D-cycloserine (20 mg/kg IP or bilateral amygdal infusion) rescued behavior
*Shank2* mutants (Won et al. 2012)	Mice	Not shown	Three chamber sociability test;deficit in sociability + D-cycloserine rescued behavior
Humans with social anxiety disorder (J. A. J. Smits et al. 2020a, b)	Humans	Not shown	D-cycloserine during exposure therapy improved social anxiety symptoms

In addition to direct SRR manipulation, d-serine levels are also controlled by amino acid transporters. Wang and colleagues used chronic social defeat stress (CSDS) to evaluate whether alanine-serine-cysteine transporter 2 (ASCT2/Slc1a5) is involved in depressive-like behavior (Wang et al. 2017). Ten days following CSDS, social interaction was evaluated by placing mice in an open field arena with a cage containing an aggressor mouse in the center, and time spent in a surrounding “interaction zone” was recorded; it was found that stressed mice spent less time in the interaction zone than control mice. High-performance liquid chromatography (HPLC) showed decreased d-serine in the hippocampus of CSDS mice, while bilateral injection of d-serine into the hippocampus rescued social interaction. In addition, ASCT2 acetylation was enhanced after CSDS, corresponding to increases in mRNA and protein expression primarily in the CA1 and CA3 regions of the hippocampus. Reducing ASCT2 expression via bilateral injection of shRNA into the hippocampus increased d-serine levels and restored social interaction. This suggests that, while ASCT2 can perform bidirectional transport of D-serine, it likely negatively regulates d-serine levels in the hippocampus after CSDS via enhanced uptake, thus contributing to social impairments in this model. Future studies exploring the role of other amino acid transporters with more abundant expression in the forebrain, such as Slc1a4 and Slc7a10 (Tapanes et al. 2022), will be critical in further elucidating the effect of D-serine release on social behavior.

Many studies have suggested that NMDAR neurotransmission influences social behavior (reviewed in (Zoicas and Kornhuber 2019)), where d-serine plays an important modulatory role. *Grin1*^*D481N*^ mutant mice, which have reduced NMDAR activity, exhibit schizophrenia-like symptoms, including impairments in both phases of the three-chamber social interaction test. However, this deficit was rescued in mice with a double *Daao*^*G181R*^ and *Grin1*^*D481N*^ mutation, indicating that enhanced d-serine can rescue NMDAR hypofunction (Labrie et al. 2010). It is important to note that in this model, *Grin1*^*D481N*^ mutant mice had a fivefold reduction in affinity at the GMS; however, in another model of NMDAR dysfunction, *Grin1* hypomorph mice, which have a 90–95% reduction of NR1 subunit expression (Mohn et al. 1999), exhibited sociability deficits that could not be rescued by d-serine due to loss of the GMS binding site (Hanks et al. 2013a, b). In addition, the Balb/c mouse strain has enhanced sensitivity to the NMDAR antagonist MK-801 relative to various strains (C57BL/6, AKR, DBA/2, Swiss-Webster (Billingslea et al. 2003; Burket et al. 2013; Deutsch et al. 1998, 1997)) and decreased sociability that can be rescued by acute injection of d-serine (Jacome et al. 2011). d-cycloserine, a d-serine analog, has been shown to ameliorate social behavior in various ASD models by restoring NMDAR signalling pathways (Won et al. 2012; Wu et al. 2018). Recently, it was found that systematic administration or direction infusion of d-cycloserine into the prefrontal cortex rescued preference for social novelty in adult neuroligin 3 R451C knockin mice, a model of ASD. Notably, intraperitoneal injection of d-cycloserine for two weeks during adolescence (P31-45), a period when the onset of NMDAR hypofunction is observed, similarly restored social novelty preference in adult mice (Cao et al. 2022), implicating d-cycloserine as a long-term treatment for social behavior. Furthermore, maternal infection during prenatal development is associated with the development of schizophrenia later in adulthood (Nagai et al. 2012). Polyriboinosinic-polyribocytidilic acid (polyI:C), a synthetic analog of double-stranded mRNA, was used to investigate the effects of d-serine and NMDARs on schizophrenia-like behavior following prenatal immune challenge (Nagai et al. 2012). At 10 weeks of age, male mice were individually housed for two days, then assessed for social interaction when an intruder mouse was introduced into their home cage. PolyI:C-treated mice had significantly less interaction time than saline-treated mice, which was restored by a single injection of d-serine 30 min prior to behavioral testing. d-serine administration had no significant effect on control mice, and l-serine had no effect on either polyI:C or saline-treated mice. Pretreatment with MK-801 prior to d-serine administration reduced the effect of d-serine on social interaction time, suggesting that prenatal polyI:C treatment interferes with the development of NMDAR-dependent signaling pathways. Together, these studies illustrate the critical role of d-serine in rodent social behavior, where decreased levels of d-serine lead to deficits in social interactions, likely through the hypoactivity of downstream NMDARs.

### Humans

d-Serine levels in the CSF of people with major depressive disorder (MDD) are negatively correlated with disease severity (Ishiwata et al. 2018), and both d-serine serum and CSF levels are reduced in people with schizophrenia (Bendikov et al. 2007; Hashimoto et al. 2005, 2003; Ohnuma et al. 2008). Furthermore, postmortem studies of people with schizophrenia have identified an increase in DAAO (Madeira et al. 2008) as well as a reduction in SRR (Labrie et al. 2009a, b) in brain tissue. Several clinical trials have investigated the effect of d-serine administration, either alone or in combination with antipsychotics, in schizophrenia (D'Souza et al. 2013; Ermilov et al. 2013; Heresco-Levy et al. 2015, 2005; Kantrowitz et al. 2016, 2018, 2010; Lane et al. 2005, 2010; Tsai et al. 1998, 1999; Weiser et al. 2012), recently reviewed in de Bartolomeis et al. (2022). While a breakdown of changes in specific measures is not provided, some studies have reported improvements in the negative symptoms of schizophrenia when d-serine was added to typical antipsychotic treatment (Ermilov et al. 2013; Heresco-Levy et al. 2005; Kantrowitz et al. 2010; Tsai et al. 1998), which include assessments of social withdrawal and avoidance. In individuals at high risk for schizophrenia, oral administration of d-serine alone significantly improved negative symptoms (Kantrowitz et al. 2015). Interestingly, the addition of d-serine to clozapine, a partial NMDAR agonist (Tsai et al. 1999), or atypical antipsychotics (Lane et al. 2010) did not improve negative symptoms, suggesting that the effectiveness of d-serine depends on the mechanism of action of co-administered therapies. Furthermore, as negative affective symptoms in schizophrenia are highly comorbid with MDD, clinical findings have prompted the investigation of the role of d-serine in anti-depressive effects (MacKay et al. 2019). In addition, clinical trials have established a positive role for d-cycloserine in the treatment of MDD (Henter et al. 2021) and social anxiety disorders (Smits et al. 2020a, b), suggesting the need for further studies on the therapeutic potential of d-serine.

#### d-Serine and sleep

d-serine was shown to be important for sleep regulation via mutations that affect d-serine levels and administration of NMDAR antagonists in fruit flies and rodents. Sleep in fruit flies is described by the duration, intensity, latency to sleep, recovery after sleep deprivation, and arousal level (Dai et al. 2019). The Drosophila Activity Monitoring System (DAMS) measures sleep by using infrared beams to detect and quantify fly movement over time; if the beam is not broken for five or more minutes, fly activity is counted as sleep (Beckwith and French 2019; Nakagawa et al. 2022). This method allows for the quantification of the frequency and duration of sleep bouts, or how often a fly sleeps and wakes up. In rodents, patterns of coordinated changes in electroencephalogram (EEG) and electromyogram (EMG) readings can be used to measure sleep (Naylor et al. 2011), as EEG signals reflect the electrical activity of the brain and provide information about the level of neuronal activity. High-frequency EEG activity (> 12 Hz) is typically associated with wakefulness, while low-frequency activity (< 5 Hz) is often seen during sleep. On the other hand, EMG measures the electrical activity of muscles, where a low EMG signal may indicate sleep while a high amplitude EMG signal may indicate active movement (Naylor et al. 2011). EEG and EMG recordings can also be used to distinguish non-rapid eye movement (NREM) from rapid eye movement (REM) sleep. NREM sleep is characterized by a decrease in body temperature, heart rate, respiration, and EEG and EMG activity. In contrast, REM sleep is characterized by an increase in body temperature, heart rate, and respiration, and EEG and EMG activity (Mondino et al. 2021). In humans, self-report can be used in addition to electrophysiological data. For example, sleep diaries can be used to establish baseline sleep patterns (Dutcher et al. 2021), and questionnaires can be used to account for details including the duration, latency, efficiency, and disturbances of sleep as well as the use of sleeping medication and daytime dysfunction to assess overall sleep quality (Buysse et al. 1989).

##### Fruit flies

D-serine has been implicated in sleep regulation using transgenic models, where SRR knockout flies were found to have significantly reduced nighttime sleep duration, increased latency to sleep, and elevated arousal rates that can be rescued by exogenous d-serine administration (Dai et al. 2019) (Table 3). Mutating serine hydroxymethyltransferase, an enzyme involved in the synthesis of l-serine, resulted in reduced sleep duration that could be reversed through either l-serine or d-serine supplementation (Dai et al. 2019). Conversely, sleep duration was increased in *daao* hypomorphic mutant flies, which have reduced catabolism of d-serine, supporting the role of d-serine in promoting sleep (Nakagawa et al. 2022). d-Serine administration through feeding increased sleep duration in a dose-dependent manner in WT flies (Nakagawa et al. 2022). Interestingly, astrocyte-like glia cells and not neurons showed an increase in sleep-wake bouts but no change in sleep duration  (Nakagawa et al. 2022). However, d-serine failed to restore reduced sleep duration in *nmdar1* hypermorphic mutants (Nakagawa et al. 2022) and *nmdr1* knockout flies (Dai et al. 2019), suggesting that d-serine regulates and promotes sleep in flies via NDMAR signaling.

**Table 3:** Effect of d-serine on sleep activity

Model: Genetic/treatment	Organism	d-serine	Sleep
SRR KO (Dai et al. 2019)	Drosophila	↓	Reduction in sleep duration during the dark phase No change in sleep duration during the light phase Administration of d-serine (2.9 g/L) rescued phenotype
SRR knockdown (Astrocyte-like glial cells) (Nakagawa et al. 2022)	Drosophila	↓	No change in sleep duration Increase in sleep–wake bouts
SHMT mutant (Dai et al. 2019)	Drosophila	↓	Reduction in sleep duration in both the dark and light phase Administration of either l/d-serine (2.9 g/L) rescued deficits
DAAO -dko (Dai et al. 2019)	Drosophila	↑	Increase in sleep duration in both the dark and light phase
*Daao1* mutants (Nakagawa et al. 2022)	Drosophila	↑	Increase in sleep duration
WT D-serine administration (≥ 50 mM) (Nakagawa et al. 2022)	Drosophila	↑	Increase in sleep duration
SRR KO (Aguilar et al. 2021)	Mice	↓	No change in either non-REM/REM Sleep No change in wake state in both number and duration
WT (Papouin et al. 2017)	Mice	Hippocampus Wake dark (active) phase ↑ light (sleep) phase, ↓	Changes in d-serine during the dark and light phase
Social Anxiety Disorder (Dutcher et al. 2021)	Humans	↑	d-Cycloserine (50 mg) did augment the effect of exposure therapy on sleep quality
Control (Alizadeh Asfestani et al. 2018)	Humans	Not shown	d-Cycloserine (175 mg) increased the effect of sleep on memory retention

*SHMT* Serine hydroxymethyltransferase, *DAAO -dko* d-amino acid oxidase double knock-out

Interestingly, a recent study has shown that changes in nighttime sleep duration in *srr* mutants can be rescued by the reintroduction of SRR specifically into intestinal epithelial cells (Dai et al. 2019). This result shows that intestinal d-serine signaling is important in the homeostatic regulation of sleep, suggesting a novel role of the intestine in sleep regulation (Dai et al. 2019).

##### Rodents

Similar to flies, d-serine reduces the sedative response induced by alcohol in rodents (Lockridge et al. 2012), and one study suggests that d-serine levels oscillate during wakefulness and sleep (Papouin et al. 2017). Extracellular recordings of NMDAR-mediated field excitatory post-synaptic potentials from the hippocampus of mice sacrificed at different times during the day show that NMDARs are saturated with d-serine at the end of the dark (active) phase, which rapidly declines to non-saturating levels in the light (sleep) phase, and progressively builds up again throughout the dark phase (Papouin et al. 2017) (Table 3). However, in another study employing SRR knockout mice, there were no significant changes in the percentage of time in each sleep/wake vigilance state, the average bout length, or the average bout frequency in SRR knockout relative to WT mice (Aguilar et al. 2021) (Table 3). Further, characteristics of sleep spindles (i.e., spindle density, amplitude, median and mean duration, median frequency), which are EEG presentations of non-REM sleep, were also unchanged in SRR ablated mice (Aguilar et al. 2021) (Table 3). However, this could be due to the fact that studies were performed on mice that were single-housed while tethered to an EEG recording, which might affect natural sleeping patterns. To address this possibility, it will be important to conduct more studies on the effect of d-serine using wireless telemetry transmitters that enable continuous measurements of EEG, EMG, locomotor activity, and subcutaneous temperature in freely moving rodents (Missig et al. 2018). Furthermore, future studies employing conditional SRR knockout mice will be critical, as germline knockout mice could have compensatory mechanisms during development that mask the role of d-serine in adult sleep patterns.

##### Humans

In a study of 51 healthy participants, a 175 mg dosage of d-cycloserine increased the effect of sleep on memory retention. Specifically, it was found that the learning of new words was better after sleep than wakefulness (Alizadeh Asfestani et al. 2018), suggesting that d-cycloserine can improve the ability of sleep to aid in the retention of new information (Table 3). In individuals with social anxiety disorder (SAD), d-cycloserine (50 mg) did augment the effect of exposure therapy on sleep quality. It is worth noting that the study did not examine the direct impact of d-cycloserine on sleep and that the sleep (quality and time) data was self-reported. (Dutcher et al. 2021) (Table 3).

## Conclusion

**Fig. 1:**
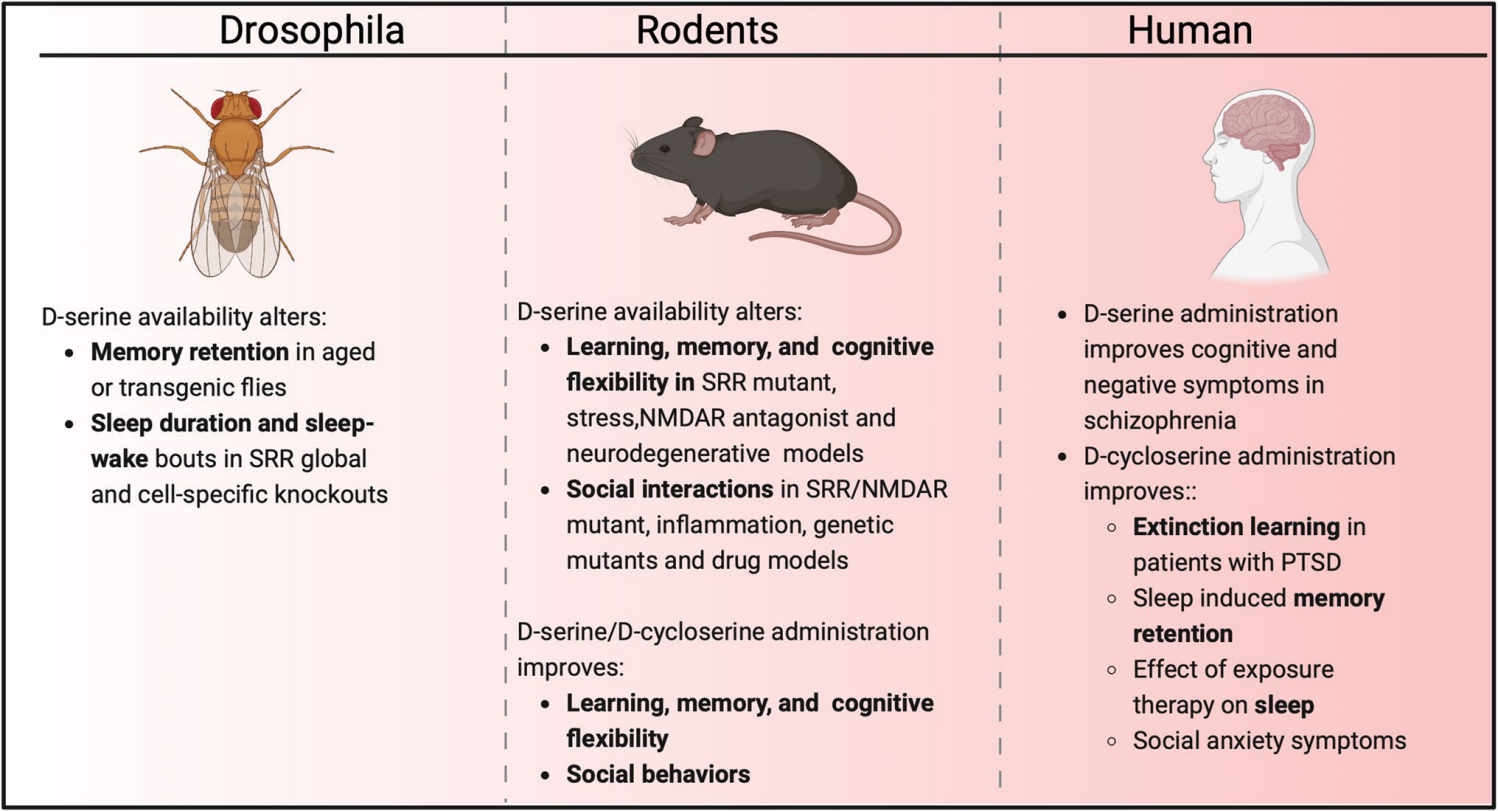
Summary showing the effect of d-serine and d-cycloserine on cognition, sleep and social behavior across species. Image was created in Biorender

Model systems are an essential tool in the development of new pharmacotherapeutics targeting sociability and cognition, despite challenges in translating higher-order cognitive processes. While no disease model can fully reflect human behavior, it can effectively reproduce underlying molecular and cellular pathologies. This review summarizes findings on the effect of d-serine on cognition, social interaction, and sleep in different species (Fig. 1). Administration of d-serine has shown promising results in rescuing learning and memory impairments induced by SRR knockout and environmental factors in flies and rodents. In humans, d-serine supplementation to antipsychotic treatments for schizophrenia enhanced cognitive flexibility. Furthermore, d-serine or d-cycloserine enhanced fear extinction in both mice and humans with PTSD. In mice, there was a time-dependent role of d-serine in learning and memory, as shown by the different effects in immediate, delayed or post-retrieval extinction (Inoue et al. 2018); it will, therefore, be important to consider the timing of d-serine administration in future clinical trials.

Although there were some variations in the detection of deficits in sociability (Labrie et al. 2009a, b) versus preference for social novelty (Aguilar et al. 2021; Matveeva et al. 2019), the effects of d-serine on social behavior were generally consistent across rodent studies. Notably, systemic administration of d-serine was sufficient to rescue social impairments across various models, including SRR ENU mutagenesis (Labrie et al. 2009a, b), CSDS (Wang et al. 2017), Balb/c strain mice (Jacome et al. 2011), and PolyI:C injection (Nagai et al. 2012), implicating d-serine as a possible therapeutic for social withdrawal and avoidance. In humans, little to no clinical trials have directly assessed the effect of d-serine on social behavior. However, preliminary studies show that d-cycloserine is able to ameliorate symptoms of social anxiety, suggesting further exploration of the role of d-serine on social behavior is warranted. While d-serine was found to regulate sleep in flies, further research is needed in rodents using wireless telemetry transmitters that enabled continuous measurements of EEG, EMG, locomotor activity, and subcutaneous temperature to compare to human data (Missig et al. 2018). Also, more in vivo studies that measure absolute d-serine levels during sleep and wakefulness will be vital. In humans, studies showing the beneficial effect of d-cycloserine on sleep-induced memory retention indicate that it may directly play a role in sleep, given that REM sleep is essential for memory consolidation.

A common limitation across many studies is that NMDAR function is not evaluated to determine whether the effect of d-serine on behavior is directly linked to NMDAR signalling. However, various studies have shown that SRR knockout mice have a reduction in NMDAR currents and NMDAR-induced excitatory postsynaptic potentials (EPSPs) in the hippocampus and dentate gyrus (Balu et al. 2013, 2016; Basu et al. 2009; Benneyworth et al. 2012; Dallerac et al. 2021; Perez et al. 2017). Furthermore, postsynaptic deletion of SRR regulates NMDAR-dependent synaptic plasticity (Wong et al. 2020), and neuronal-specific SRR knockout reduced LTP in the naïve hippocampus whereas astrocytic SRR knockout prevents TBI-induced deficits in hippocampal LTP (Perez et al. 2017). These findings demonstrate a critical role for d-serine in NMDAR function, suggesting that d-serine’s effect on behavior is likely through downstream signalling. This possibility is supported by the fact that the ameliorating effect of d-serine on social interaction in PolyI:C treated mice was antagonized by pretreatment with an NMDAR antagonist, MK-801 (Nagai et al. 2012). Furthermore, reducing catabolism of d-serine in *Daao*^*G181R*^ and *Grin1*^*D481N*^ double mutants rescued social behavior relative to *Grin1*^*D481N*^ mutants. Studies employing various models of ASD have demonstrated a direct link between the effect of d-cycloserine administration on social behavior and NMDAR signalling, suggesting a similar mechanism for d-serine (Cao et al. 2022; Won et al. 2012; Wu et al. 2018). Moreover, in sleep studies, d-serine increased sleep duration in WT flies but did not rescue reduced sleep in nmdar1 knockout flies (Dai et al. 2019) and NR1 hypomorphic mutant flies (Nakagawa et al. 2022). Together, these findings demonstrate a relationship between d-serine, behavior, and NMDAR activity that warrants further investigation to exclude the role of NMDAR-independent mechanisms.

Overall, these studies highlight the potential of d-serine as a therapeutic target for improving sociability, cognition, and sleep in various contexts. Further studies employing conditional, cell-specific knockout models to explore the downstream effects of d-serine on NMDAR signalling pathways will be critical to better understand the mechanisms underlying the effects of d-serine and to further establish its clinical applications.

## Data Availability

Not applicable.

## Abbreviation

SR: Serine racemase
DAAO: D-Amino acid oxidase
